# Current Concepts of Fracture-Related Infection

**DOI:** 10.1155/2023/4839701

**Published:** 2023-04-25

**Authors:** Si-ying He, Bin Yu, Nan Jiang

**Affiliations:** ^1^Division of Orthopaedics and Traumatology, Department of Orthopaedics, Southern Medical University Nanfang Hospital, Guangzhou 510515, China; ^2^Guangdong Provincial Key Laboratory of Bone and Cartilage Regenerative Medicine, Southern Medical University Nanfang Hospital, Guangzhou 510515, China

## Abstract

Currently, fracture-related infection (FRI) still represents great challenges in front of orthopaedic surgeons, despite great advances that have been achieved regarding its diagnosis and treatment. Although both FRI and prosthetic joint infection (PJI) belong to osteoarticular infections and share similarities, FRI displays unique characteristics. Diagnosis of FRI is sometimes difficult owing to the nonspecific symptoms, and treatment is usually tricky, with a high risk of infection recurrence. In addition, the long disease course is associated with a significantly elevated risk of disability, both physically and psychologically. Moreover, such a disorder still poses heavy economic burdens to the patients, both personally and socially. Therefore, early diagnosis and reasonable treatment are the key issues for increasing the cure rate, decreasing the risks of infection relapse and disability, and improving the life quality and prognosis of the patients. In this review, we summarized the present concepts regarding the definition, epidemiology, diagnosis, and treatment of FRI.

## 1. Introduction

As one of the most frequent types of bone and joint infection, fracture-related infection (FRI) often refers to infection following fractures or during the treatment process for fractures. At present, FRI remains to be a catastrophic disease for both clinicians and patients despite great efforts spared in its various fields. Although FRI shares similarities with prosthetic joint infection (PJI), another type of osteoarticular infection, it has unique features. One of the typical characteristics of FRI is its high heterogeneity. To be specific, on the one hand, although FRI diagnosis is established based on different tools, presentations of clinical symptoms and imaging tests, serum levels of inflammatory biomarkers, microorganism culture results, and even treatment strategies differ among different patients with FRI. Thus, the clinical efficacy and prognosis vary. On the other hand, the pathogenesis of FRI is complex, the occurrence of which depends on interactions between extrinsic factors and intrinsic factors. The extrinsic factors mean external factors, which include but are not limited to injury type, injured site, contamination degree of the wound, and even the prophylaxis and treatment methods, while intrinsic factors are host factors, especially for the immune status of the patients at the time of injury. Also, lifestyle and comorbidities are also internal factors relating to FRI development. As mentioned above, although the current situation of FRI is still unoptimistic, early and accurate diagnosis, with reasonable and standard treatment, is a vital measure to increase the cure rate, decrease the recurrence risk, restore limb function, and improve the life quality of patients. Here, we summarized recent findings regarding the definition, epidemiology, diagnosis, and treatment of FRI, aiming to provide updated evidence for clinical reference.

## 2. Definition

In 2018, Morgenstern et al. [1] conducted an international survey with an 11-item questionnaire among orthopaedic trauma surgeons regarding the definition of FRI. Outcomes from 2,327 responses revealed that there was a lack of a definite FRI definition, which necessitates a consensus. Also in 2018, Metsemakers et al. [2] conducted a systematic review focusing on the FRI definition and totally analyzed 100 randomized controlled trials (RCTs). They found that only 2% of the studies cited a validated FRI definition, with 28% using a self-defined definition. With the support of the Arbeitsgemeinschaft für Osteosynthesefragen (AO) Foundation and the European Bone and Joint Infection Society (EBJIS), experts from nine countries achieved a consensus for determining FRI, including confirmatory criteria and suggestive criteria [3]. Later in 2019, the Chinese expert published a consensus regarding the definition of fracture-device-related infection (FDRI) [4]. Then in 2020, the international expert group updated the 2018 diagnostic criteria for FRI, including six confirmatory criteria and six suggestive criteria [5].

## 3. Classification

Currently, there still lacks specialized classification system for FRI, the classification of which primarily consults the classification systems for osteomyelitis (OM). According to the duration from fracture or fracture intervention to the onset of infection, FRI is classified as early (shorter than 2 weeks), delayed (2–10 weeks), and late (over 10 weeks) infections [3]. The Cierny–Mader (C-M) classification [6] for OM is often selected, especially for those in the chronic stage. In 2017, Hotchen et al. [7] conducted a systematic review to summarize the classification systems for long bone OM, and they totally identified thirteen systems. After analyzing advantages and disadvantages of each system, they recommended that the following four aspects should be emphasized when classifying OM: (1) bone involvement, (2) antimicrobial resistance patterns of the causative pathogens, (3) coverage of soft tissue, and (4) host status. Based on this theory, the authors proposed the B.A.C.H classification and assessed this system, and they concluded that such a system can be applied accurately by users with different clinical backgrounds [8].

## 4. Epidemiology

### 4.1. Incidence and Risk Factors

Whether FRI occurs depends on multiple factors, which is classified as external factors and internal factors. External factors, known as environmental factors, include but are not limited to injury type and degree, injury site, pathogen virulence, and prophylaxis and treatment methods. However, even geographical location and seasonal factor can affect the occurrence of FRI [9]. Internal factors, known as host factors, primarily refer to the immune status, lifestyle, and comorbidity of the patients. In general, the average incidence of FRI ranges from 1% (closed fractures) to over 30% (open fractures) [10], with a maximum rate of 55% [11].

Incidence and risk factors of FRI differ among different fractured sites. It was reported that the FRI incidence of distal femoral joint fracture fixation was 1.5%, with open fractures, obesity, smoking, and diabetes as risk factors [12]. With respect to the tibial plateau fractures, the average FRI rate was 7%, ranging between 2.1% and 11.1% [13–15]. The risk factors of infection secondary to the tibial plateau fractures were open fractures, high-energy injuries, and smoking. Regarding the ankle fractures, the incidence of infection was approximately 6%, with obesity and alcohol overuse as risk factors [16]. As for the calcaneal fractures, the incidence of deep tissue infections was 3%. The risk factors for such a group of fractures included open fractures, high-energy injuries, American Society of Anesthesiology (ASA) grades 3 and above, and intraoperative hypothermia (<36°C) [17].

Regarding the intramedullary nail (IMN), a Brazilian study [18] showed that the infection rate following IMN for femoral and tibial diaphyseal fractures was 8.59% at 3 months, which increased to 11.8% at 12 months. Previous application of external fixators and requirement for muscle or skin flap repair were found to be risk factors of such infections [18]. As for the IMN for open fractures, Whiting et al. [19] reported that the overall incidence of IMN infection for open tibial fractures was 12%, with infection rates for type I, II, III A, III B, and III C by Gustilo–Anderson classification as 5.1%, 12.6%, 12.5%, 29.1%, and 16.7%, respectively. The risk factors included severe soft tissue injury, delayed IMN, delayed wound closure, and fracture in the distal location [19].

### 4.2. Healthcare Cost

FRI is a catastrophic complication, which not only brings physical and psychological harms to the patients but also aggravates the economic burdens to their families, even in the developed countries, such as Germany [20]. Outcomes of recent studies revealed the heavy economic consequences from different perspectives.

Jiang et al. [21] reported that the median direct healthcare cost of patients with posttraumatic OM was 4.8-fold higher than those without infection ($10,504 vs. $2,189, dollars) in China. Potentially influencing factors of the cost for OM included use of external fixator, external fixator type, infection location, and infection-related injury type. Similarly, Parker et al. [22] found that patients with deep surgical site infection (SSI) following open fractures in the lower limb had increased health and social care costs than those without infection (mean difference, £1,950, pounds) in the UK. Meanwhile, SSI seriously impaired health-associated quality of life. In Belgium, Iliaens et al. [23] observed that the direct hospital-related cost of patients with FRI was eight times that of the non-FRI patients (€47,845 vs. €5,983, euros). In addition, the median indirect cost of the FRI patients was about four times that of the non-FRI patients (€77,909 vs. €19,706). Likewise, the FRI patients had worse physical function and poorer pain score. In a recent retrospective study, Barrés-Carsí et al. [24] conducted a comparative analysis regarding the healthcare resource and cost of infection following tibial fractures in a Spanish cohort. Outcomes showed that the total hospitalization cost for patients with infection increased from €7,607 to €17,538. Meanwhile, patients with infection had significantly longer or higher hospital length of stay, readmissions, and mean operating theatre time.

Although increasing evidence has demonstrated FRI-related heavy economic burdens, it should be noted that the actual cost is usually underestimated. The primary reason is that indirect cost or potential cost has not been calculated, such as cost of lost labor and transportation fee to and from the hospital.

## 5. Diagnosis

Diagnosis of FRI is established based on comprehensive considerations of the medical history, clinical signs and symptoms, imaging tests, and laboratory tests (serological levels of the inflammatory biomarkers, pathogen identification strategy, and histological test). As mentioned above, the diagnostic criteria of FRI were proposed by an international consensus in 2018 [3] and updated later [5], including confirmatory criteria and suggestive criteria. A recent study [25] validates the diagnostic criteria of FRI, and the authors confirmed the excellent diagnostic discriminatory value of the confirmatory criteria. For suggestive criteria, specificities of over 95% were obtained for clinical signs of fever, wound drainage, and local redness. This implies satisfying efficacy of such criteria for FRI diagnosis.

### 5.1. Medical History, Clinical Signs, and Symptoms

Patients with FRI often have a definite history of trauma and orthopaedic surgery. It was previously believed that whether typical signs or symptoms occur largely depends on the infection stage and pathogen virulence. To be specific, acute phase FRI is primarily caused by highly virulent pathogens while late or chronic infections are mainly caused by less virulent pathogens. However, a 2022 study [26] failed to find enough evidence to support the belief that more virulent pathogens are associated with early infections while less virulent pathogens are often related to the late infections. Therefore, they concluded that the relevance of classifying FRI by time since injury remains unclear from perspective of microbiology. Considering the conclusions were derived from a single report with limited sample size, multicenter studies are warranted.

### 5.2. Imaging Tests

The auxiliary diagnostic values of the imaging tests mainly rest with the following three aspects: (1) providing more evidence for determining whether there exists FDRI; (2) providing visual details for FRI, such as infection range and distributions of sinus and fistula, for making a surgical plan; and (3) evaluating the status of fracture reduction, the situation of fracture healing, and the stability of the internal fixation [27].

Recent research hotspots mainly focused on the nuclear medicine tests, including bone scintigraphy (BS), leukocyte scan (LS), positron emission tomography (PET), and fusion imaging (SPECT/CT, PET/CT, and PET/MRI). BS displays a high sensitivity (89%–100%) but a low specificity (0%–10%) for FRI diagnosis, the outcomes of which are easily influenced by resent trauma and surgery. Thus, some researchers do not recommend BS as a routine test for FRI diagnosis [27]. Regarding the LS (LS), one superiority of this approach is its accuracy not affected by trauma or surgery [28]; however, it is time-consuming and laborious [29], with less accuracy for diagnosis of infection in axial skeleton [30, 31]. In a recent study, Lee and Kim [32] evaluated the feasibility of bone SPECT/CT for surgical planning of patients with chronic OM (COM) in the lower limbs. They found that bone SPECT/CT is a feasible strategy in assisted diagnosis of COM, which can also be applied among patients with recent trauma and surgery, as well as among those with implants. With respect to the FDG-PET/CT, outcomes from previous studies revealed that its sensitivity and specificity for diagnosing FRI ranged from 65% to 94% and from 76% to 100%, respectively [27]. Lemans et al. [33] found that the risk of misdiagnosis for patients suspected of bone infection using the FDG-PET/CT within 1 month after surgery was as high as 46%. However, such a risk was reduced to 7% between 1 month to 6 months after surgery. Thus, it is not suggested using FDG-PET/CT to detect acute-phase FRI. According to a recent expert consensus on clinical application of FDG-PET/CT in the diagnosis of infection and inflammation [34], the recommended level of FDG-PET/CT for diagnosis of peripheral OM is Level III (may be significant for clinical diagnosis and treatment), with evidence level of Level *C* (expert consensus, small studies, retrospective studies, and registries), while the recommended level for PJI diagnosis is Level II (which is likely to be significant for clinical diagnosis and treatment), with evidence level as Level *B* (single RCTs or large non-RCTs). This implies that more studies with high level of evidence should be conducted to assess the role of FDG-PET/CT for assisted diagnosis of FRI. In a prospective case series, Hulsen et al. [35] noted that PET/MRI was able to provide the same diagnostic information for COM as PET/CT did, but PET/MRI was able to display additional information of the soft tissue.

In brief, nuclear medical tests have both advantages and disadvantages. There is still lack of sufficient evidence to conclude which one is the optimal in detecting FRI. In the future, innovative techniques should be developed, such as imaging techniques being able to detect bacterial biofilms, bone viability, and drug-resistant bacteria [36].

### 5.3. Inflammatory Biomarkers

The white blood cell (WBC) count, erythrocyte sedimentation rate (ESR), and C-reactive protein (CRP) are traditional and classic serum inflammatory markers. Due to multiple influencing factors and differences in half-life times, their diagnostic values in FRI differ. WBC usually rises to the highest level at day 1 to day 3 after surgery and is back to normal within 4 to 6 days [37]. ESR usually increases to the highest level at day 7 to day 11 after surgery and decreases to normal after 6 weeks gradually [38]. CRP usually goes up to the highest level on the second day following surgery and returns to normal after 2 weeks [39]. In a recent systematic review and meta-analysis, van den Kieboom et al. [40] summarized current evidence regarding the value of WBC, ESR, and CRP for late FRI diagnosis. Outcomes based on six studies indicated that CRP achieved the highest sensitivity (77%), with ESR in the top of specificity (79.3%). Nonetheless, the authors concluded that all the three indicators are insufficiently accurate to diagnose late FRI, which can be applied as a suggestive sign of infection. Aside from focusing on single detection outcome of the serological levels of the biomarkers, dynamic monitoring of changes in the levels of such biomarkers is also of great significance for judging whether there exists infection. However, other potential influencing factors, such as recent surgery, stress, and hypersensitivity, should be excluded first [41]. As CRP displays the highest sensitivity among the three traditional biomarkers, it is frequently used to monitor whether infection occurs after surgery. However, it is not recommended to detect serum CRP level in the first three days after surgery, as a recent study [42] failed to find any differences of the CRP levels between patients with and without complications in the first three days postoperatively. Similarly, Shin et al. reported that CRP was able to determine the presence of systemic infections after internal fixation of intertrochanteric fractures in the aged as early as the fifth postoperative day [43]. Therefore, as described in the expert consensus, repeated acquisition of CRP levels is suggested in suspicion of early-stage infection. If serum CRP level continuously increases from 4th to 7th day after surgery, a high probability of FRI should be considered after exclusion of infection in other systems or persistent systemic inflammatory stress status of the patient [4]. Aside from surgery, bacterial type and virulence can also affect the levels of the inflammatory markers, meaning that high-virulence and drug-resistant bacteria are often associated with higher levels [44].

In recent years, increasing number of studies reported the potential roles of some novel biomarkers in the FRI diagnosis. In 2017, Shahi et al. [45] reported that serum D-dimer is a promising marker for PJI diagnosis. Later in 2019, Wang et al. [46] also found that serum D-dimer level may be useful indicator for evaluation of infected nonunion, with sensitivity and specificity as 75% and 91.2%. In addition to D-dimer, Zhao et al. [47] noted that interleukin-6 (IL-6) had similar diagnostic value for FRI, with comparison to ESR and CRP. Other analyzed indicators for FRI diagnosis included platelet count to mean platelet volume ratio [48], wound alpha defensin [49], and cluster of differentiation (CD) 64 [50]. It is interesting that even altered gut microbiota can be considered as an auxiliary indicator for FRI diagnosis [51]. Nonetheless, considering the limited number of such reports, more investigations should be performed in future to better evaluate the efficiency of these biomarkers in assisted diagnosis of FRI.

### 5.4. Microorganism Culture and Identification

Phenotypically indistinguishable pathogens detected from at least two independent specimens from deep tissues have been listed as one of the confirmatory criteria for FRI [5]. Intraoperative sample culture is still the gold standard for detection of FRI-associated pathogens. However, the positive rate remains far from satisfying. According to a recent multicenter study [52], the positive rate of traditional sample culture was only 50.8%. It is known that outcome of such a traditional culture strategy is influenced by multiple factors, such as culture condition, recent antibiotic use and surgery, and selection of the specimens. A recent study [53] found that even the number of samples collected for culture could affect the outcomes. Based on the findings, they suggested that at least 5 deep tissue specimens are recommended for culture. In order to increase the detection rate, several recommendations had been proposed [27], including that antibiotics should be stopped for at least two weeks before surgery, intraoperative antibiotics are suggested to be administrated immediately after sampling, samples should be ideally collected from the implant-bone interface, and separate, aseptic surgical instruments should be used for each sample collection. In addition, different culture media should be considered to cover both aerobic and anaerobic pathogens, such as brain heart infusion broth, MacConkey agar, Brucella blood agar, blood plate, and chocolate plate. It is advised that the culture time is generally for 7 days but can be extended to 14 days in suspicion of slow-growing strains, such as *Cutibacterium acnes* [27, 54].

Recently, different adjunctive strategies for pathogen identification were reported, aiming at increasing the detection rate. Bellova et al. [55] reported the efficiency of sonication in the diagnosis of FRI, and outcomes of 230 retrieved implants revealed that sonication of fracture devices may be a useful adjunct for FRI. In a meta-analysis focusing on the diagnostic accuracy of sonication fluid aspiration for FRI, Ahmed et al. [56] reported that the sensitivity and specificity of the sonication method were 86% and 98%, with the tissue sample culture as 98% and 38%, respectively. Thus, they suggested combining the two methods. In addition, Jiang et al. [57] introduced a novel method for pathogen identification, referred to as “implant surface culture,” which is based on the theory that there may exist residual bacterial biofilms attached to the implant surfaces. They found that this novel method can detect additional FRI-associated pathogens, which cannot be detected by the traditional method. Also, the culture time of the implant surface culture was shorter than the traditional method. Finally, they concluded that this method is a useful adjunct to the traditional method for detection of pathogen causing FRI. Later, their team reported using the similar technique, “devascularized bone surface culture,” for identification of COM-related pathogens [58]. Outcomes revealed that compared with the traditional culture, such a devascularized bone surface culture displayed a relatively higher positive rate (74.5% vs. 58.8%) and a significantly shorter culture time (1 day vs. 3 days). Apart from the abovementioned strategies, there are still authors who reported culture from the reamer-irrigator-aspirator (RIA) system, also with satisfying outcomes [59].

Although the new emerging methods display encouraging outcomes, their diagnostic efficiency is still required to be evaluated by more future studies. Currently, intraoperative standard sample culture is the gold standard for detection of pathogens causing FRI.

### 5.5. Molecular Biological Identification

Molecular biology primarily refers to amplification of bacterial DNA through polymerase chain reaction (PCR) technology. Although previous study had reported that the PCR technique showed superiority in detection of low-virulent bacteria [60], a systematic review indicated that PCR failed to show obvious advantages over the traditional method in diagnosis of FRI [61]. Considering that only two studies were included for analysis, cautious attitude should be taken towards the conclusions. Future PCR technology should focus on how to reduce the false positive rate, improve diagnostic sensitivity and accuracy, and provide detailed information regarding drug sensitivity.

### 5.6. Histopathological Examination

According to the updated expert consensus for FRI diagnosis, visible microorganisms and presence of over 5 NPs/HPF by histological analysis are two confirmatory criteria for FRI diagnosis [5]. Nonetheless, the detailed diagnostic criteria are still under investigation. In 2018, Morgenstern et al. [62] analyzed the role of quantitative histological analysis in FRI diagnosis. Based on analysis of 156 patients, the authors found that the sensitivity and specificity were 80% and 100% using the cutoff value of 5 NPs/HPF for diagnosis of infected nonunion. Thus, they concluded that the existence of over 5 NPs/HPF has a positive predictive value for FRI of 100%, while the absolute absence of any NPs is always an indicator of an aseptic nonunion. Later in 2020, Sybenga et al. [63] proposed an algorithm-derived strategy for histological diagnosis of OM. In this method, they firstly classified OM into five categories, including acute OM, acute and chronic OM, chronic OM, chronic active OM, and chronic inactive OM. Then, they assigned a histologic load score to different levels of criteria with reference to fifteen generally agreed-on histologic features of OM and obtained a final score, named as Jupiter score. After analyzing scores from 263 patients, they concluded that diagnosis of OM can be established for Jupiter score of ≥6, with scores ≤4 basically indicating exclusion of infection. Other diagnostic clues should be referred to in case of the score of 5. This scale provides a coordinated and unified quantitative standard for the pathological diagnosis of OM, but its diagnostic efficacy remains to be assessed by more clinical studies.

## 6. Treatment

The general treatment principles for FRI include radical debridement, implant handling, systemic and local antibiotics, reconstruction defects of bone and soft tissues, and functional recovery. Multidisciplinary teams, including surgeons, infectious diseases specialists, pharmacists, and microbiologists, are recommended to improve the treatment efficacy [64, 65]. Selection of the specific treatment methods should consider many factors, such as infection site and duration, pathogen type and virulence, host immunity and requirements, and expectations of the patients. In a 2019 systematic review, Bezstarosti et al. [66] summarized the present evidence regarding the treatment of FRI. Outcomes of 93 studies with 3,701 patients demonstrated that the second-stage surgery was dominant (54%) of the treatment strategy. The overall cure rate was 93%, with cure rate following the first-stage surgery as 85%. The infection recurrence rate was 9%, with the limb amputation risk as 3%.

### 6.1. Radical Debridement

Radical debridement is one the most effective ways to reduce the bacterial load of the infected tissues, which is also the key to lower the risk of infection recurrence [67]. The debridement styles may differ among different C-M anatomical classifications and infection sites. For example, the RIA system is often applied for type I intramedullary infection [68], while for type III localized calcaneus infection, an “eggshell-like” debridement has been proved to be effective [69]. Despite the debridement styles, the basic principle is still complete removal of all the necrotic and devascularized tissues. With respect to debridement rinse capacity, loading method (high/low pressure), and additives, based on an updated expert review, the optimal rinse capacity of debridement remains inconclusive, and disputes still exist regarding the delivery method. It is definite that it is not advised to add any antibiotics or surfactants (e.g., Castile soap and benzalkonium chloride) to the rinse solutions, but preservatives, such as chlorhexidine, can be considered [70].

### 6.2. Implant Handling

Removal or retention of the implants requires comprehensive consideration of multiple factors, including the implant-bone structure stability, infection location and duration, host physiological status, pathogen type and virulence, soft tissue conditions, and the possibility of radical debridement [54]. In the Chinese expert consensus on FDRI treatment [4], implants are suggested to be removed in any of the seven situations, including patients addicted to drug or smoking, compromised immunity which cannot recover in a short time, open fracture, IMN fixation, unsatisfactory fracture reduction or unstable fixation, poor soft tissue condition or insufficient wound coverage, and difficult-to-treat bacteria. In addition, the implant should also be considered for removal in case of acute compartment syndrome, especially with soft tissue necrosis and infection.

As infection duration directly determines the state of the bacterial biofilm, it is previously believed that time from fracture fixation is an important indicator to decide whether implants can be retained. As for early or acute FRIs, the success rate of DAIR (debridement, antibiotics, and implant retention) surgery can reach 90% [71, 72]. In a recent systematic review, Morgenstern et al. [73] analyzed the influence of infection duration on outcomes of the DAIR surgery for FRI management. Outcomes revealed that the success rates of DAIR surgery for acute, delayed, and late FRIs were 86% to 100%, 82% to 89%, and 67%, separately. Finally, they concluded that infection duration is not the only factor that should be considered for FRI treatment. For the detailed strategy, Qiu et al. [74] reported coating the plate with antibiotic cement for the DAIR surgery and obtained satisfying outcomes.

### 6.3. Systemic Antibiotics

Appropriate systemic antibiotic use is another effective way to reduce the risk of infection relapse for FRI treatment in addition to debridement. Recent research hotspots mainly focused on administration route, treatment duration, and emerging antibiotics.

Clinical efficacy of oral administration of antibiotics for FRI treatment has gained wide attention. In 2019, a multicenter, open-label, and parallel-group RCT [75] was published in the NEJM, which compared oral vs. intravenous antibiotics for osteoarticular infection (the OVIVA trial). Outcomes of 1,054 participants revealed that patients that received oral antibiotics achieved similar efficacy as those by intravenous approach. Although the incidence of serious adverse events was similar between the two, the risk of catheter complications was much lower in the oral group. Thus, they concluded that oral antibiotic was noninferior to intravenous antibiotic for management of bone and joint infections. Meanwhile, their subsequent analysis indicated that oral antibiotics for the treatment of osteoarticular infection for the first six weeks were less costly and do not cause detectable differences with comparison to the treatment intravenously [76].

In early 2020, with the support of the AO Foundation, the EBJIS, the Orthopaedic Trauma Association (OTA), and the PRO-IMPLANT Foundation, on behalf of the FRI Consensus Group, nine experts from the Europe and the United States proposed recommendations for systemic antibiotic therapy of FRI [77]. In this study, seven specific surgical strategies are described, and decision for the duration of systemic antibiotics mainly depends on implant handling strategy, culture outcome, and possibility of anti-biofilm antibiotics. The recommendation fills the gap in related treatment fields, but the specific implementation process is relatively complex. In addition, long-term antibiotic therapy for some specific situations may reduce the compliance of the patients and increase the risk of side effects. The current treatment strategies proposed are based on expert opinions, and RCTs are necessary to evaluate the efficacy, especially for the necessity of long term of antibiotics. A 2019 RCT showed that no significant difference was found regarding the rates of clinical or microbiological remission between patients that received systemic antibiotics for four weeks and six weeks after debridement and removal of the implants for FRI [78].

It is known that bacteria in the biofilm state can be up to 1,000 times more resistant to antibiotics than those in the planktonic state [79]. Therefore, how to effectively eradicate the biofilm is the key to decrease the risk of infection recurrence for FRI treatment, especially for those with implant retention. In suspicion of biofilm-related infection, biofilm-active antibiotic agent is suggested, which had been certified of rifampicin combinations against staphylococci and fluoroquinolones against Gram-negative bacteria [77]. Aside from the traditional antibiotics, recent studies also evaluated the efficacy of some novel antibiotic agents. In a 2019 systemic review, Telles et al. [80] assessed the efficacy of daptomycin for the treatment of osteoarticular infections and PJIs. Outcomes revealed that the cure rates for device-related and non-device-related infections were 70% and 78%, respectively. In addition to daptomycin, recent studies [81] also reported satisfying efficacy of dalbavancin for management of OM.

### 6.4. Local Antibiotics

As mentioned above, radical debridement is the key to reduce the risk of infection relapse; however, a dead cavity is often formed after debridement. If the cavity is not treated effectively, the local environment is likely to cause the “resurgence” of bacteria and may eventually lead to infection recurrence. Therefore, dead space management also influences the treatment efficacy. Local antibiotic implantation is an effective way to eliminate the dead space in addition to assisting in eliminating the residual bacteria.

Also, in early 2020, the experts from the FRI consensus group published recommendations for local antimicrobial and dead space management methods for FRI [82]. As summarized in this study, the frequently locally used antibiotics are gentamicin, tobramycin, vancomycin, and clindamycin. Other antibiotics that had been reported for local use include cefazolin, daptomycin, erythromycin, polymyxin, linezolid, amphotericin, voriconazole, and amikacin. Altogether ten items of key recommendations had been proposed, and of the key recommendations, several should be paid special attention to. For example, clinical evidence for application of the naked antibiotics (e.g., vancomycin powder) remains limited for the treatment of FRI. Also, local and systemic toxicity should be alerted among certain patients. Higher doses of antimicrobials may display better efficacy for infection control; however, they may cause side effects. Aside from antibiotics, different types of antibiotic carriers are summarized, including autograft, allograft, polymethylmethacrylate (PMMA), ceramic products, poly(D,L-lactide), collagen sponges, and hydrogels. Furthermore, the nonantibiotic antimicrobial methods against infection, including silver and bacteriophages, are also introduced, providing new insights into local strategies for FRI treatment.

In addition to the expert recommendations, the Oxford University Bone Infection Center had proposed an “Oxford protocol” for the treatment of bone infections based on different C-M anatomical classifications. Local implantation of calcium sulfate with antibiotics is recommended for intramedullary (type I), localized (type III), and diffused (type IV) infections, while soft tissue coverage is recommended for the superficial infection (type II) [83]. As a representative of degradable ceramic products, calcium sulfate has been widely used in clinical practice. Recent studies confirmed the satisfying efficacy of local calcium sulfate with antibiotics in the treatment of bone infections, including among the pediatric patients [69, 84–86]. In addition to calcium sulfate, other types of antibiotic carriers have been reported, such as collagen sponge [87], porous alumina ceramic [88], and bioactive glass [89]. However, the number of such studies is still limited, which needs to be evaluated by more future studies.

### 6.5. Bone Defect Reconstruction

Autogenous bone graft remains the gold standard for treatment of bone defects shorter than 2.5 cm [90]. As for the large segmental bone defects, the selection of reconstruction strategies depends on multiple factors, such as experience of the surgeons, site and size of the bone defect, patient comorbidity, and compliance. The most frequently used methods to reconstruct segmental bone defect include the Ilizarov technique, the Masquelet technique, and free vascularized fibular grafting technique.

For the Ilizarov technique, recent research hotspots mainly focus on the efficacy of double-level bone transport and acute shortening in bone defect reconstruction. Several recent investigations have reported the advantages of double-level bone transport, such as shorter time with external fixation, faster bone healing time, fewer complications, and better function recovery, with comparison to the single-level bone transport [91–93]. In addition, recent studies also focused on the efficacy of acute shortening followed by lengthening. In a 2020 meta-analysis, Wen et al. [94] compared the efficacy between acute shortening and bone transport for management of infected tibial defect. Outcomes of five studies demonstrated that acute shortening could reduce the treatment period, while bone transport could lower the risk of bone grafting. Subsequently, a 2022 systematic review [95] summarized the efficacy of acute shortening for management of open tibial fractures with bone and soft tissue defects. Based on an analysis of twenty-four articles, the authors concluded that acute shortening is an alternative to microsurgical techniques to solve defects of osseous and soft tissues.

The Masquelet technique, also known as the induced membrane technique, is another effective way to repair large bone defects. Recently, professor Masquelet AC himself reviewed the history and development of this technology, clarified the indications, discussed its biological and molecular basis, and provided the key tips for optimal success [96]. In this study, a total of nine recommendations were proposed and five achieved evidence grade *B* (fair evidence), with the remaining four items as grade *C* (poor-quality evidence). The five grade *B* recommendations are as follows. (1) The Masquelet technique is an effective strategy to reconstruct bone defects. (2) Radical and meticulous debridement of the devascularized bone at both stages is essential for a success. (3) Preservation and incision of the induced membrane at the second stage is crucial for bone graft containment and its successful remodeling. (4) The addition of antibiotics to the cement spacer is effective for producing a viable induced membrane. (5) The optimal time for the second stage of surgery is between 4 and 8 weeks after the first stage of surgery.

Regarding the clinical efficacy of the Ilizarov technique vs. the Masquelet technique, outcomes of a current meta-analysis [97] revealed that, compared with the Ilizarov technique, the Masquelet technique displayed superiorities in lower hospitalization cost, shorter final union time, shorter time to full weight bearing, lower risk of complications, and better quality of life after surgery. Considering the limited number of the included studies, as well as their limited evidence level, more future investigations are warranted.

In addition to the abovementioned two technologies, free vascularized fibula grafting technique is also an effective approach to repair large bone defects. Antonini et al. [98] evaluated using this technique for the treatment of bone defect among patients with localized and diffused OM. After analyzing the results from 18 patients, they concluded that vascularized fibula graft is an effective way to reconstruct bone defect; however, a well-trained multidisciplinary team is required to dispose the high risk of potential complications, such as stress fractures. Also, Adam et al. [99] introduced using this technique to repair bony defects in pediatric patients following resection of tumor and neurofibromatosis. Outcomes based on 25 patients confirmed that clinical efficacy of such a technique is definite; however, the perioperative complication risk was 32%. Recently, with the emergence of three-dimensional (3D) printing technology, personalized and precise repair and reconstruction are no longer out of reach. In a prospective study, Liu et al. [100] reported using the 3D-printed porous Ti6Al4V scaffolds to repair critical diaphyseal defects of the lower limbs and achieved satisfying postoperative functions and low complication rates.

### 6.6. Repair of Soft Tissue Defects

Timely and effective coverage of soft tissue defects is critical for both prevention and treatment of FRI. The detailed strategies to repair soft tissue defects require considerations of multiple factors, such as microsurgical experience of the surgeons, patient age, smoking status, comorbidity, and location and size of the soft tissue defect. In a 2019 systematic review, Bezstarosti et al. [66] summarized methods to repair the defect of soft tissue, and outcomes showed that free flaps (39%), skin grafts (21%), and rotational flaps (11%) were the more frequently selected strategies. In a recent systematic review and meta-analysis, Dow et al. [101] compared the efficacy of free muscle flaps with free fasciocutaneous flaps for reconstruction defects in the lower limbs following trauma. Outcomes showed similar efficacy regarding the incidences of total flap failure, reoperation, and limb salvage between the two methods.

Negative pressure wound therapy (NPWT) can provide much convenience in the treatment of both open fractures and FRI. However, controversy still exists regarding the efficacy of this technique. According to the outcomes of two recent RCTs (WOLLF [102] and WHIST [103]) published in the JAMA, NPWT is not recommended for treatment of severe open fractures. Also, incisional NPWT is not suggested for fractures in the lower limbs associated with major trauma. The studies uncover the possible limitations of NPWT technology, but its advantages should not be fully denied. Regarding the role of NPWT in FRI therapy, a 2021 systematic review [104] indicated that there was still lack of strong evidence to support the use of NPWT as a definitive treatment for FRI. Similarly, in a recently published retrospective cohort study, Sweere et al. [105] found that delayed wound closure with NPWT increased the risk of infection recurrence among patients with soft tissue defects following FRI treatment. Thus, they suggested that NPWT should be considered only as a few days of necessity to bridge the period until the establishment of definitive wound closure.

## 7. Prevention

FRI is a catastrophic complication of fractures for both patients and clinical physicians, and prevention is better than cure. In order to effectively lower FRI incidence, comprehensive understanding of the pathogenesis of this disorder is necessary. The development of FRI is related to both extrinsic factors and intrinsic factors. Extrinsic factors include but are not limited to fracture type and location, microorganism virulence, and even prophylaxis strategy. Recent studies [106, 107] indicated that local antibiotic use as a preventive measure could effectively decrease the risk of infection. In addition, Alamanda and Springer [108] proposed twelve modifiable risk factors for the prevention of PJIs, which may be also applicable for FRI.

Aside from the modifiable risk factors, there are still unmodifiable variables, with genetic predisposition as a representative. Growing evidence has suggested that single-nucleotide variations may also participate in the development of FRI, such as polymorphisms located in the *vitamin D receptor* gene (rs7975232 and rs1544410) [109], the *interferon*-*γ* gene (rs2430561) [110], and the *interleukin* genes (rs16944, rs1143627, rs1800796, and rs4251961) [111]. Therefore, such data of Genome-Wide Association Study can be used to screen population in a higher risk to develop FRI, and preventive measures should be taken in advance, aiming at reducing the risk of FRI occurrence.

## 8. Future Perspectives

Although FRI aroused attention only in recent years, it has achieved rapid and great progress in diagnosis and treatment, which are still not enough. In the future, more clinical investigations, with high level of evidence, should be conducted to increase the rate of early and accurate diagnosis and improve the treatment efficacy. Moreover, in-depth fundamental research should also be performed to uncover the pathogenesis of this disorder more comprehensively.

## Data Availability

No data were used to support this study.
